# Exploring the Potential of Broadly Neutralizing Antibodies for Treating SARS-CoV-2 Variants of Global Concern in 2023: A Comprehensive Clinical Review

**DOI:** 10.7759/cureus.36809

**Published:** 2023-03-28

**Authors:** Sai Dheeraj Gutlapalli, Vijay Durga Pradeep Ganipineni, Sumanth Danda, Daniel Fabian, Ikpechukwu J Okorie, Jananthan Paramsothy, Tharunjan Kailayanathan, Rushaniya Umyarova, Cinthya Aviles, Sameer Krishna Prasad Garlapati, Derek Ugwendum, Jay Nfonoyim

**Affiliations:** 1 Internal Medicine, Richmond University Medical Center Affiliated with Mount Sinai Health System and Icahn School of Medicine at Mount Sinai, Staten Island, USA; 2 General Medicine, SRM (Sri Ramaswamy Memorial) Medical College Hospital and Research Center, Chennai, IND; 3 General Medicine, Andhra Medical College/King George Hospital, Visakhapatnam, IND; 4 Internal Medicine, Katuri Medical College and Hospital, Guntur, IND; 5 Internal Medicine, Andhra Medical College/King George Hospital, Visakhapatnam, IND; 6 Pulmonary and Critical Care, Richmond University Medical Center Affiliated with Mount Sinai Health System and Icahn School of Medicine at Mount Sinai, Staten Island, USA

**Keywords:** coronavirus disease 2019, anti-sars-cov-2 antibodies, covid-19 prophylaxis, omicron variant, intravenous immunoglobulin (ivig)

## Abstract

In the aftermath of the coronavirus disease 2019 (COVID-19) pandemic, the world is still seeing outbreaks of COVID-19 infections as of 2023, especially in populations that have been adequately vaccinated. This situation across the globe gives rise to important questions regarding the efficacy of current treatments and the real rate of mutations in the COVID-19 virus itself which can make the currently available treatments and vaccines obsolete. We have tried to answer a few of those questions and put forth some new questions of our own. Our efforts in this paper were directed towards understanding the utilization of broadly neutralizing antibodies as a treatment for COVID-19 infection with a particular focus on the Omicron variant and other newer variants. We gathered our data from three major databases: PubMed, Google Scholar, and Cochrane Central Register of Controlled Trials (CENTRAL). We have screened 7070 studies from inception till March 5, 2023, and gathered 63 articles that were relevant to the topic of interest. Based on the existing medical literature regarding the topic of interest and also based on our own personal and clinical experience treating COVID-19 patients across the multiple waves in the United States and India since the beginning of the pandemic, we have concluded that broad neutralizing antibodies could be an effective option for treatment and prophylaxis for current and future outbreaks of COVID-19 including the Omicron variant and newer variants. Further research, including clinical trials, is required to tailor optimal dosages, prevent adverse reactions/side effects, and develop treatment strategies.

## Introduction and background

There are seven coronaviruses known to infect or afflict humans [1]. Alpha-coronaviruses (α-CoVs) such as human coronavirus (HCoV)-229E and HCoV-NL63 are among these, whereas the others are beta-coronaviruses (β-CoVs) such as OC43, severe acute respiratory syndrome coronavirus-1 (SARS-CoV-1), HKU1, Middle East respiratory syndrome coronavirus (MERS-CoV), and SARS-CoV-2 [2]. The emergence by the end of 2019 of the new coronavirus SARS-CoV-2, which causes coronavirus disease 2019 (COVID-19), resulted in worldwide human infections [3]. The COVID-19 pandemic underlined the need for better treatments and vaccines.

SARS-CoV-1, the culprit responsible for the 2002-2003 outbreak of severe acute respiratory syndrome (SARS), utilizes the same host cell receptor as SARS-CoV-2: angiotensin-converting enzyme 2 (ACE2) [3]. The spike (S) protein of SARS-CoV-2 is composed of two subunits, S1 and S2, and it is essential for receptor identification and cell membrane fusion [3,4]. The S1 subunit possesses a receptor-binding domain (RBD) that interacts with the host ACE 2 receptor, whereas the S2 subunit facilitates viral cell membrane fusion by generating a six-helical bundle via the two-heptad repeat domain [4]. SARS-CoV-2 appears to maintain portions of the SARS-CoV-1 spike glycoprotein (S protein), which interacts with ACE 2 [3]. The S protein sequence of SARS-CoV-2 was matched with those of SARS-CoV-1, MERS-CoV, and common-cold coronaviruses [3]. The majority of troubling mutations have been identified in the SARS-CoV-2 spike protein, which is principally responsible for the virus's entrance into host cells [2].

The recently revealed SARS-CoV-2 Omicron variant encodes 37 amino acid changes in the spike protein, 15 of which are found in the RBD [3]. This has raised concerns about the efficacy of existing vaccines and therapeutic interventions against the virus and has sparked a debate regarding the potential need to develop new treatments and vaccines to address the new mutations. The utilization of SARS-CoV-2 neutralizing antibodies (nAbs) is an appealing therapeutic approach for COVID-19 treatment and prevention, particularly in patients with compromised immune systems, unvaccinated or vaccine-hesitant individuals, and instances where vaccines exhibit lower efficacy against certain viral strains [4-7]. The primary mechanism of action of therapeutic nAbs is the prevention of viral entry into host cells, in addition to potentially aiding in the elimination of infected host cells through Fc-mediated effector functions, ultimately leading to decreased viral load in vivo [8]. Administration of nAbs during early infection or as prophylaxis has been shown to significantly decrease the occurrence of hospitalization and mortality [8]. It should be noted that nAb therapy is not intended for severe COVID-19 cases that require hospitalization [9]. With few minor side effects, such as diarrhea, reported in approximately 1% of COVID-19 patients following nAb-based therapy, no major adverse effects have been observed [7,9].

The spike protein of the virus is the primary target for most of the currently available neutralizing monoclonal antibodies, but the rapid evolution of the virus and the emergence of new variants has led to the need for alternative strategies. One such promising strategy is the use of broadly neutralizing antibodies (bnAbs); bnAbs that target conserved regions of viral spikes have emerged as a potential strategy for treating and preventing β-CoV infections. In this literature review, we explore the potential of bnAbs for the emerging variants of coronavirus.

Methods

The literature search was conducted using databases such as PubMed, Google Scholar, and Cochrane Central Register of Controlled Trials (CENTRAL). Keywords used included “broadly neutralizing antibodies”, “bnABs”, “S2 spike protein”, “monoclonal antibodies”, “mAB”, “COVID-19”, “SARS-Cov2”, “beta-coronaviruses”, “coronavirus”, And “Omicron variant”. Studies were included if they evaluated bnAbs for coronaviruses, characterized their molecular basis and efficacy, and explored their potential applications in passive antibody treatment and vaccine design.

## Review

**Figure 1:**
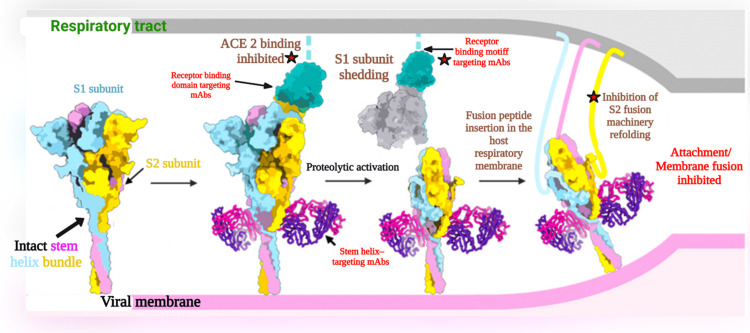
Illustration of action of neutralizing mAbs at various stages of SARS-CoV-2 entry ACE 2: angiotensin-converting enzyme 2; S1: spike protein subunit 1; S2: spike protein subunit 2; mAbs: monoclonal antibodies; SARS-CoV-2: severe acute respiratory syndrome coronavirus 2 Image credit: Ganipineni V and Gutlapalli S

Monoclonal antibodies represent a category of therapeutics that have garnered attention for their potential in the prevention and treatment of COVID-19. Typically, these agents are developed by identifying B cells specific to the pathogen in question from individuals who have recently recovered from an infection or by immunizing genetically engineered mice with a humanized immune system and harvesting effective antibodies from them [10]. Once identified, the genes responsible for encoding immune globulin heavy and light chains are retrieved, followed by their expression to produce monoclonal antibodies with singular activity against a predetermined target, such as the spike protein of the coronavirus. In contrast to convalescent plasma, which consists of polyclonal antibodies found in the serum of recovering COVID-19 patients, monoclonal antibodies (mAbs) are highly specific and targeted [11]. The actions of the various neutralizing monoclonal antibodies were clearly at the different stages of SARS-Cov-2 entry and were elaborately explained in recent studies [12]. Figure 1 details the action of neutralizing mAbs at various stages of SARS-Cov-2 entry.

**Figure 2:**
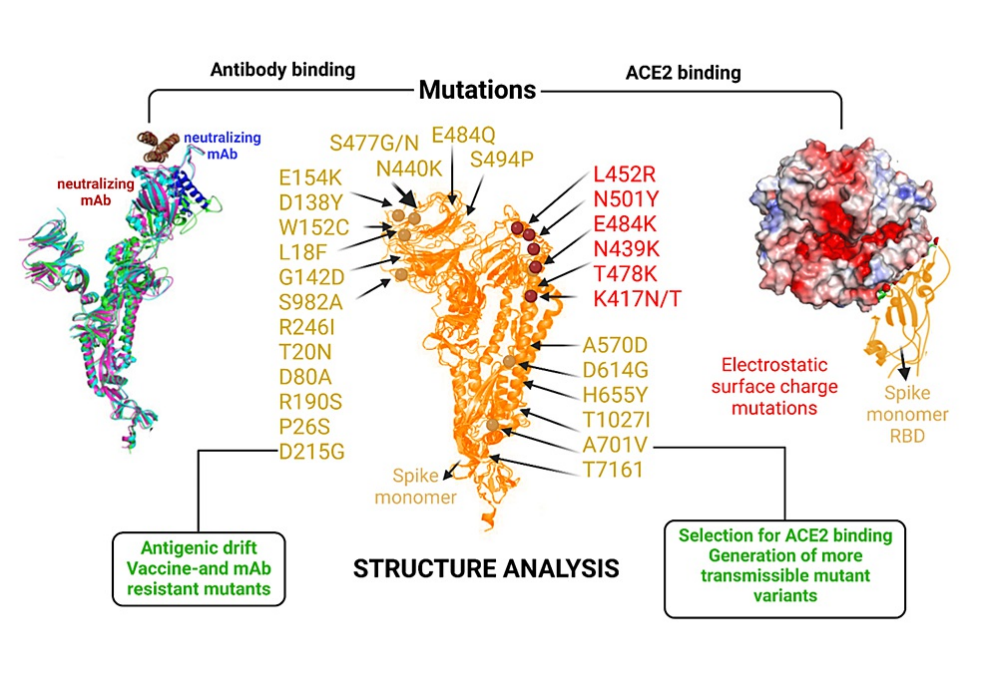
Illustration of the consequences of SARS-CoV-2 antigenic variation ACE 2: angiotensin-converting enzyme 2; mAbs: monoclonal antibodies; SARS-CoV-2: severe acute respiratory syndrome coronavirus 2; RBD: receptor binding domain Image credit: Ganipineni V and Gutlapalli S

SARS-Cov-2 antigenic mutation has been thoroughly studied in multiple studies [13]. Figure 2 illustrates the consequences of the antigenic variations of SARS-Cov-2.

Figure 2 displays the locations of mutations on the spike monomer protein, as well as their potential outcomes. Antigenic drift can result from a cluster of mutations that alter epitope configuration, potentially leading to vaccine and mAb resistance. Positive selection can also occur, with certain mutations in the receptor binding domain of the spike monomer associated with an increased likelihood of viral attachment.

One of the reasons for the success of SARS-CoV-2 vaccines is considered to be the ease of generating nAbs targeting the immunodominant epitopes on the RBD of the S1 subunit of the spike protein [14-19]. The identification of RBD-targeting nAbs has resulted in the classification of six clusters (I-VI) based on the analysis of epitopes derived from available RBD-nAb complex structures [20]. The spike protein has four categories (Group I-IV) of amino acids involved in ACE 2 binding based on their conservation and essential roles in binding [20]. Group I is composed of six identical residues, while Group II has five homologous residues [20]. Group III comprises five conditionally altered residues, while Group IV encompasses five highly diverse residues [20]. The mutations in the first two groups of amino acids are strictly limited, indicating their vital roles in maintaining protein affinity for ACE 2 or proper folding, while the substitutions in the last two groups are more tolerant and may even enhance ACE 2 binding, frequently observed in various variant of concern (VOCs) [20]. Table 1 provides an overview of current neutralizing mAbs against SARS-CoV-2. However, it is important to note that the antibodies' effectiveness can vary depending on the virus's evolution and mutation patterns. Moreover, the spike protein is a dynamic molecule that can undergo structural changes depending on its interaction with host factors or immune system components, which can affect the antigenicity and immunogenicity of the protein. As such, studies are needed to understand the complexity of the virus-host interplay and to identify potential targets for safe and effective treatments or vaccines.

**Table 1:** . Summary of the current state of neutralizing monoclonal antibodies against SARS-CoV-2 CDC: Centers for Disease Control and Prevention; FDA: United States Food and Drug Administration; mAbs: monoclonal antibodies; VBM: variant being monitored; VOC: variant of concern; RBD: receptor binding domain; RBM: receptor binding motifs; Eff: effective; InEff: ineffective; Red: reduced; Ab: antibody: SARS-CoV-2: severe acute respiratory syndrome coronavirus 2 Table Credit: Ganipineni V and Gutlapalli S

Variant name	Ab Class	Target	FDA	Source of mABs	Alpha	Beta	Gamma	Epsilon	Delta	Omicron
Current CDC Classification [21-25].					VBM	VBM	VBM	VBM	VBM	VOC
Lineage [21-25].					B.1.1.7	B.1.351	P.1	B1.427 B.1.429	B.1.6172	B.1.1.529
List of the Clinical mABs:										
Bamlavinimab [21-25].	Class2	RBD	✔	Human	Eff	In Eff	In Eff	In Eff	In Eff	In Eff
Etesvimab [21-25].	Class1	RBM	✔	Human	Red	In Eff	In Eff	Eff	Eff	In Eff
Bebtelovimab [21-25].	-	RBD	✔	-	Eff	Eff	Eff	Eff	Eff	Eff
Casirivimab [21-25].	Class1	RBM	✔	Human	Eff	Red	In Eff	Eff	Eff	In Eff
Imdevimab [21-25].	Class3	RBD	✔	Human	Eff	Eff	Eff	Eff	Eff	In Eff
Sotrovimab [21-25].	Class3	RBD	✔	Human IgG1	Eff	Eff	Eff	Eff	Eff	Eff
Tixagevimab [21-25].	-	RBM	✔	Human IgG1	-	-	-	-	-	Partial
Cilgavimab [21-25].	-	RBM	✔	Human IgG1	-	-	-	-	-	Partial
Regdanvimab [21-25].	-	RBM	✗	Human IgG1	-	-	-	-	-	Partial
Adintrevimab [21-25].	Class1&4	RBD	✔	Human	Eff	Eff	Eff	-	Eff	Eff

Researchers conducted a study to determine the frequency of in vivo nAb targets and viral mutations, using immunogenic and mutational heatmaps derived from nAb complex structures of the RBD [20]. The study observed a positive correlation between hot immunogenic sites and areas with high mutation frequencies, with certain exceptions regarding certain sites involved in ACE 2 binding [20]. Only three out of the top 10 immunogenic residues exhibited substitutions in circulating SARS-CoV-2 variants, with Q493R and Y505H being newly acquired in Omicron [20]. The hot immunogenic residues, such as F486, Y489, Y449, N487, and F456, are predominantly composed of Type I and II residues located within the ACE 2 binding sites, and mutations at these locations have not been identified among circulating variants [20]. This indicates that these residues are highly conserved and have integral roles in the interaction between the ACE 2 receptor and S protein. However, the newly acquired mutations in the Omicron variant, particularly the Q493R and Y505H, may potentially alter the viral protein conformation, leading to alterations in their binding capacity to the ACE 2 receptor. Furthermore, the most potent nAbs according to previous studies, are antibodies that target epitopes overlapping with the ACE 2 receptor binding site (RBS), divided into RBS A/class 1; RBS B, C, D/class 2 antibodies requiring minimal affinity maturation to neutralize the virus at very low concentrations (single digit ng/ml) [14,17,21,26-31].

bnAbs, as the name implies, neutralize multiple viral strains [32]. These antibodies target conserved epitopes of the virus, which means that even when the virus undergoes mutation, the targeted epitopes will still be present [33]. Conversely, non-bNAbs are specific to individual viral strains and have unique epitopes [14]. The discovery of bnAbs for HIV-1 has led to the development of vaccines that can be applied not only to HIV-1 but also to other fast-mutating viruses, such as SARS-CoV-2 [14]. In vivo protection against SARS-CoV-1, SARS-CoV-2, and MERS-CoV has been demonstrated by bnAbs, which further provide the potential for antibody-based interventions and insights for developing pan-β-CoV vaccines [14]. However, the emergence of several VOCs is due to mutations around the RBS, particularly in and around the RBS, which generate viral variants that are resistant to the majority of RBS-A/class 1 or RBS-B, C, D/class 2 nAbs [3,14,34-44]. VOCs such as Beta and Gamma, with K417N/T and E484K mutations, respectively, are known to be resistant to the vast majority of RBS-A/class 1 and RBS-B, C, D/class 2 nAbs [14,31,34,45]. The Omicron variant has been found to escape neutralization by some nAbs, which target the more conserved regions of the RBD [14,38,44]. However, the S2 region on the coronavirus spike provides alternative, relatively conserved target regions with neutralizing epitopes, which may be useful for generating SARS-CoV-2 vaccines that are effective against VOCs and pan-β-CoV vaccines [14,46-49].

The development of vaccines against diverse β-CoVs may be facilitated by the use of bnAbs that target the conserved spike S2 epitopes. Researchers have also recently identified a nAb called CC40.8 from a COVID-19 convalescent donor that targets the spike S2 stem-helix region, neutralizes SARS-CoV-2 VOCs and sarbecoviruses from clades 1a and 1b, and forms a critical component of the spike fusion machinery [14,50,51]. The study showed that the use of CC40.8 offers protection against SARS-CoV-2 infection in human ACE 2 mouse and hamster models [14,51]. A large panel of stem-helix bnAbs will be essential for developing rational vaccine strategies that induce bnAbs targeting the S2 stem-helix region through vaccination [14,52-56]. Certain stem-helix bnAbs show greater potency against clade 1a SHC014 and clade 1b SARS-CoV-2 compared to other sarbecoviruses, and the IGHV1-46 (63%) and IGHV3-23 (22%) germline gene families were significantly enriched in stem-helix antibody sequences [14,57,58]. The stem-helix bnAbs with MERS-CoV neutralization demonstrated even greater enrichment of the IGHV1-46 germline gene (78%), indicating its potential role in broader reactivity against diverse β-HCoV spikes. The CDRH3 loop lengths in the isolated stem-helix bnAbs were predominantly 10- and 11-residue-long CDRH3s compared to the human baseline reference database, and the CDRL3 loop lengths in the stem-helix bnAbs were mostly 9- and 11-residue CDRL3 loops with germline JL-gene-encoded motifs, important for epitope recognition [14]. Combining S2 bnAbs with the most appropriate RBD bnAbs might be the optimal prophylactic approach for SARS-CoV-2, particularly with the emergence of new variants such as Omicron, though further studies are needed to measure the appropriate dose for effective prophylaxis [14]. It is noteworthy that such findings are based on the works by researchers in isolating nAbs that target the stem-helix region of the spike protein and trigger cross-reactivity against various human coronaviruses and variants [14].

Researchers identified six mAbs that bind to spike proteins from all seven human-infecting coronaviruses using an epitope-agnostic approach [59]. These six mAbs were able to target the conserved fusion peptide region located adjacent to the S2' cleavage site [59]. Two of the identified mAbs, COV44-62 and COV44-79, were shown to have broad neutralization activity against α-CoVs and β-CoVs, including SARS-CoV-2 Omicron subvariants BA.2 and BA.4/5 [59]. However, the neutralization potency of these mAbs was lower than that of RBD-specific antibodies [59]. The crystal structures of the COV44-62 and COV44-79 antigen-binding fragments, when combined with the SARS-CoV-2 fusion peptide, revealed a helical structure covering the arginine residue at the S2' cleavage site [59]. In contrast, Parray and colleagues recently announced the identification of a potent mAb, P4A2, that can effectively neutralize all current circulating VOCs, including Omicron [60]. By determining the crystal structure of the P4A2 Fab: RBD complex, the residues of the RBD were identified as part of the ACE 2-receptor-binding motif (RBM), which remains unchanged in all VOCs [60]. The specificity of P4A2 for SARS-CoV-2 and its VOCs was confirmed by the pan-coronavirus pseudotyped neutralization assay [60]. Administration of P4A2 to K18-hACE2 transgenic mice provided both prophylactic and therapeutic protection against VOCs in the study [60]. These results suggest that P4A2 could serve as a potential treatment option for neutralizing currently circulating VOCs [60].

Various researchers have illustrated the mechanisms that the vast majority of mAbs go through that target the RBM by losing their in vitro neutralizing activity against Omicron, with only three of the 29 monoclonal antibodies retaining their original potency, specifically the ACE-2-mimicking S2K146 antibody [3,61]. Furthermore, some broadly neutralizing sarbecovirus mAbs can neutralize Omicron by recognizing antigenic sites located outside the RBM, including sotrovimab, S2X259, and S2H97 [3,12,62,63]. This evidence indicates that the extent of Omicron-mediated immune evasion represents a significant antigenic shift in SARS-CoV-2 [3].

Collectively, these findings indicate that mAbs that possess broad neutralization capacity and recognize conserved RBD epitopes as well as S2 across both SARS-CoV-2 and other sarbecoviruses could be critical for managing the current pandemic and preventing future zoonotic transmission events.

Future implications for COVID-19 treatment

Future research on bnAbs could involve several aspects. One area of exploration is the design of immunogens to induce protective antibody responses against the Omicron variant. Such exploration would be informed by the unique features of the antibody germline genes. Additionally, efforts could focus on enhancing the effector functions of anti-S1 and S2 bnAbs, such as antibody-dependent cellular cytotoxicity (ADCC) and complement-mediated lysis, which could provide improved protection against Omicron. Clinical trials are also necessary to evaluate the safety, efficacy, and long-term effects of treatments and vaccines based on bnAbs. Another potential area of research is investigating the optimal combination of different bnAbs. As the virus continues to mutate, it is also important to explore the impact of viral escape mutations on the efficacy of bnAbs. Researchers could study the structural characteristics and mechanisms of neutralizing activity of bnAbs, which could inform the design of more potent treatments and vaccines against SARS-CoV-2. Finally, future research could focus on identifying the optimal timing and dosage of anti-S2 bnAbs for the treatment and prophylaxis of SARS-CoV-2 and its variants

## Conclusions

Based on our analysis of the existing literature, we found that the conserved anti-S2 bnAbs and anti-S1 RBD bnAbs exhibit broad neutralizing activity against diverse range of coronaviruses, including SARS-CoV-2, SARS-CoV-1, and MERS-CoV. Structural studies revealed that the stem-helix bnAbs recognize a common hydrophobic core epitope that is conserved across β-CoVs. The isolation of a large panel of β-CoV bnAbs has provided a framework for rational vaccine design strategies that can take advantage of the germline gene features of bnAbs. Animal studies have also shown that the stem-helix bnAbs protect against diverse β-CoVs in high efficacy, suggesting their potential as stockpiled reagents for future outbreaks. Additionally, the use of S2 bnAbs, possibly in combination with the RBD bnAbs, may be optimal for SARS-CoV-2 prophylaxis. Effector functions play a critical role in the therapeutic activity of anti-S2 bnAbs despite their typically lower neutralization potencies than RBD bnAbs. Stem-helix bnAbs exhibited potent S2-mediated effector functions, including ADCC and complement-mediated lysis, which could enhance protection against Omicron. Furthermore, the analysis of some of the anti-S2 bnAbs revealed the unique features of the antibody germline genes, which could facilitate the rational design of immunogens that induce protective antibody responses against Omicron.

In conclusion, the conserved S1 RBD, S2 stem-helix, and fusion peptide regions as candidate epitopes for next-generation coronavirus vaccine and broadly neutralizing mAb development. The large panel of β-CoV bnAbs isolated may serve as a valuable resource for counteracting future outbreaks. Nevertheless, further studies, including human clinical trials, are required to evaluate the safety, efficacy, and potential long-term effects of these bnAb treatments and vaccines.
